# From awareness to integration: a qualitative interview study on the impact of digital therapeutics on physicians’ practices in Germany

**DOI:** 10.1186/s12913-025-12656-2

**Published:** 2025-04-18

**Authors:** Jennifer Kendziorra, Kirsten H. Seerig, Till J. Winkler, Heiko Gewald

**Affiliations:** 1 Chair of Information Management, University of Hagen, Hagen, Germany; 2 Copenhagen Business School, Copenhagen, Denmark; 3 Institute for Digital Innovation (IDI), Neu-Ulm University of Applied Sciences, Neu-Ulm, Germany

**Keywords:** Digital health, Digital therapeutics, German healthcare system, Physician roles, Medical practices, Qualitative research

## Abstract

**Background:**

The integration of digital therapeutics (DTx) into the German statutory healthcare system marks a significant shift in medical practice through the introduction of innovative, reimbursable digital interventions for patient care. While DTx can bridge therapy gaps and enhance patient care, the number of prescriptions is increasing slowly. This study aims to explore the challenges physicians face when integrating DTx into their treatment options and the changes this integration entails for their medical workflows.

**Methods:**

A qualitative approach was adopted, gathering data from semi-structured interviews with 46 physicians across Germany. Participants, sampled for diversity in specialty, experience, and region, were interviewed between June 2022 and August 2024. The interviews explored physicians’ knowledge, experiences, and perspectives on DTx. Data were analyzed using qualitative content analysis, combining deductive and inductive coding.

**Results:**

The introduction of DTx into physicians’ workflows impacts their practice on multiple levels. First, physicians must become aware of the new regulations and therapeutic possibilities, requiring significant information intake and time to feel adequately prepared for prescribing DTx. Second, DTx add complexity to patient assessment, as physicians must evaluate factors such as digital literacy, motivation, and cognitive conditions, necessitating a deeper understanding of their patients. Third, the integration of DTx reshapes the physician‒patient relationship, as it alters interaction dynamics, redistributes responsibilities, and poses new communication challenges. Fourth, DTx expand therapeutic options, particularly by bridging therapy delays and enabling more personalized care. Finally, the integration of DTx has the potential to drive long-term changes in physicians’ workflows and mindsets, fostering alignment with multimodal and patient-centered treatment approaches.

**Conclusions:**

DTx offer opportunities for patient care but also pose challenges, such as the development of digital skills or familiarization with new regulations, that necessitate adjustments in physicians’ workflows. Physicians are generally confronted with an innovation that demands time, reliable information on effectiveness, practical experience, and openness to adopting digital health tools as complementary treatment options. However, the reception and integration of DTx into treatment vary among physicians, reflecting individual preferences and approaches. Further research should explore these diverse adoption strategies and examine the long-term effects of DTx on care delivery and patient outcomes.

**Supplementary Information:**

The online version contains supplementary material available at 10.1186/s12913-025-12656-2.

## Background

Digital therapeutics (DTx) are an innovative and emerging component of digitalization in the healthcare sector. According to a market research report, the global market size of DTx amounted to approximately four billion US dollars in 2021 and is expected to reach approximately 23 billion US dollars by 2031 [2]. DTx have the potential to contribute significantly to the treatment of chronic pain or chronic diseases, such as diabetes [5]. Unlike other digital and mobile health (mHealth) applications (apps), such as wellness apps, the distinctive feature of DTx is that they are tailored to specific medical conditions and rely on evidence-based medicine [16, 31]. Digital therapeutics (DTx) can be defined as software-based therapeutic interventions that aim to improve the process of diagnosis, management, treatment, and prevention of diseases and injuries [7, 10].

Germany is among the first countries worldwide that has implemented a structured reimbursement model for DTx through statutory health insurance. Digital therapeutics must demonstrate positive care effects and meet specific requirements for data protection and interoperability to qualify as a “DiGA” [3]. This abbreviation stands for the German term for ‘digital health application’ and refers to prescribable DTx in Germany. To be approved for medical prescription and reimbursement by statutory health insurance, DiGAs must be listed in the DiGA directory maintained by the German Federal Institute for Drugs and Medical Devices (BfArM) [7, 9]. To be included in this directory, a DiGA must demonstrate to the BfArM that it fulfills the necessary requirements in terms of safety and functionality, data security, interoperability and user-friendliness and that it can demonstrate medical evidence [3]. In Germany, there are two ways in which patients can receive a DiGA that is reimbursed by their statutory health insurance [8]: (1) A physician prescribes a DiGA to the patient and the patient submits this prescription to the health insurance company, or (2) the patient contacts the health insurance company directly with a corresponding diagnosis, but without medical prescription, to request a preferred DiGA. In both cases, the health insurance provider verifies the patient’s entitlement and generates a prescription code for the corresponding DiGA. The patient uses this code to activate the DiGA for the specified usage period [4]. The costs of the DiGA that the patient receives through this procedure are reimbursed by the health insurance company directly to the DiGA manufacturer according to negotiated and fixed prices [13, 18]. Other countries, including Belgium, Japan and the USA, undertake similar efforts to integrate DTx into primary care or adapt the reimbursement model that was introduced by the Digital Healthcare Act in Germany in 2019 [10, 12]. The DiGAs listed in the directory of the BfArM cover a range of various medical conditions, such as diabetes, adiposity or substance abuse disorders. Therefore, General Practitioners (GPs) in particular are able to prescribe DiGAs from various medical fields such as orthopedics and mental health to their patients, which allows them to bridge waiting times for therapeutic treatment and provide holistic patient care [6].

In addition to the positive effects mentioned on patient care, DTxs are also subject to debate and pose significant potential challenges in terms of data protection and security, equal access to treatment options, regulation and clinical evidence [5]. Although it is stipulated that prescribable DTx must demonstrate medical evidence, concerns have been raised regarding inadequate blinding in clinical trials, the considerable potential for bias and high drop-out rates within the studies [21, 22]. In Germany, DTx are also temporarily approved for prescription even though they did not yet prove any positive treatment effects in ongoing studies [21]. The short study duration, which is imposed by the requirements of the BfArM, is likewise criticized [9]. Another significant point of criticism, which is cited by the National Association of Statutory Health Insurance Providers, is the nontransparent cost structure of reimbursable DTx in Germany. In the first year of approval, DTx manufacturers are granted producer prices that often considerably exceed the average reimbursement rates for DTx [13]. On the other hand, the great financial pressure on DTx manufacturers during the development phase and the lack of funding opportunities are also seen critically [27], and some manufacturers have already filed for insolvency.

Due to the newly developed regulatory framework and slowly increasing prescription rates [13], this provides an optimal context for investigating how these tools impact the healthcare workforce. Among other stakeholders, such as patients, health insurance companies or DTx manufacturers, physicians and psychotherapists play a distinct role when it comes to DTx, as they operate both as prescribers and gatekeepers in the process [8, 26]. They are pivotal in deciding whether DTx are effectively integrated into treatment processes [8]. This makes them particularly interesting for a study on DTx adoption. In addition, the implementation of digital technologies in healthcare and the shift toward more patient-centered care also entail new levels of complexity and require healthcare professionals to reflect and adjust their traditional roles and responsibilities [14].

While significant progress has been made in understanding the integration of digital tools into the healthcare system, limited research has explored how digital therapeutics impact physicians’ roles and responsibilities, change their work practices, and investigate their perceptions of this new technology [6]. Previous research has mainly focused on understanding the key influencing factors for the acceptance and adoption of DTx by patients [19, 30] and physicians [1, 6, 7], as well as the benefits and challenges associated with DTx use and implementation. Among the frequently reported benefits of DTx are more flexible access to medical care, patient empowerment, and increased patient engagement [7]. Patients’ health competences and knowledge are enhanced by allowing them to self-manage and monitor their health conditions [7]. Further advantages are the possibility of overcoming treatment gaps through DTx [6] and thus addressing unmet therapeutic needs, as well as the time savings achieved through new medical treatment options [6, 7].

Despite their potential, DTx are still prescribed to a limited extent in Germany [8]. In the healthcare system in general, technological innovations tend to be adopted more reluctantly than in other industries [23]. This resistance to change has led to the slow integration of new technologies such as DTx into widespread medical care as well as existing treatment pathways and workflows [12, 23]. The reasons for this include the substantial amount of time required for physicians to familiarize themselves with DTx [6] as well as the necessary adaptation of workflows to ensure seamless integration into the treatment processes [7]. In addition, interoperability issues as well as usability and technical challenges remain further significant obstacles to DTx implementation [12].

Furthermore, the high costs of DTx and the lack of financial compensation for physicians’ efforts to integrate them into their treatment plans and acquaint themselves with them constitute major barriers to the widespread utilization of DTx in Germany [7, 12]. Although physicians receive lump-sum remuneration for medical and psychotherapeutic services in the context of DTx-related follow-up tasks [15], this remuneration does not apply to the initial prescription or previous training efforts and familiarization with DTx [11]. However, financial incentives are necessary for DTx uptake [31], to ensure that physicians are financially compensated for the time and effort spent on training and familiarization with DTx and to prevent additional costs from arising at their expense [11]. In most cases, physicians do not feel sufficiently informed about this treatment option or question its clinical evidence and benefits [6]. The skepticism may be further reinforced by the high drop-out rates among patients using DTx and their low adherence [6, 8, 12]. Legal uncertainties regarding liability and recourse claims as well as insecurities regarding data protection represent further obstacles [7]. Another reason why physicians are skeptical about the implementation of DTx and why DTx uptake is relatively low is the weakening of therapeutic relationships and the partial replacement of interpersonal physician‒patient contact [12]. Clinicians fear that face-to-face consultations will be diminished using DTx and that the physician may be replaced as the first point of contact. This applies in particular to consultation-based therapies such as psychotherapy, where mutual trust plays an important role during treatment [12]. Many practitioners therefore advocate embedding digital solutions such as DTx into in-person medical treatment rather than replacing it entirely [11].

In addition to skepticism, digital health literacy among the healthcare workforce is low in many cases, which is a diametrical obstacle to the consistent use of DTx [17]. To facilitate the widespread use of DTx, these skills must therefore be enhanced [17]. This is also the conclusion of a questionnaire study examining the digital health literacy of German physicians before the onset of the Covid-19 pandemic and advocating for the integration of digital health literacy into medical curricula. Most of the 93 physicians surveyed had limited or insufficient knowledge about the use of eHealth technologies, especially in terms of data security [20]. As the study claims representativeness regarding the age of the participants (approximately 40% were between 30 and 45 years and 32% between 45 and 60 years), the findings seem to be applicable to the German medical profession at large [20].

While current research clearly highlights the relevance of the topic, it has mainly identified and presented a variety of relevant factors and consequences without analyzing and understanding the associated changes in depth. However, the implementation of DTx into primary care implies a change in the way physicians understand their role and the interaction between physicians and patients, which requires deeper insight into the dynamics. A qualitative study on GPs’ attitudes toward the prescription of mHealth applications revealed that many physicians are afraid of a “dehumanization” of medicine and a curtailment of their professional autonomy and decision-making ability [26]. However, even if GPs are rather skeptical about DTx, they believe that this development is unavoidable [26]. Accordingly, DTx need to be integrated into medical treatment plans and the understanding of healthcare professionals’ roles. For physicians, the adaptation of DTx involves changing their routine workflows and addressing new challenges. In their scoping review, in which Wattanapisit et al. [32] examine whether mHealth applications can replace GPs in the future, the authors conclude that medical tasks cannot be completely substituted by digital solutions but can be partially replaced. This applies to diagnostics, health promotion and decision-making support, among others. It is therefore recommended that GPs develop expertise in the use and integration of digital health applications into their treatment processes [32]. Building on the insights of previous research and in the context of DTx, an innovative and spreading digital health innovation, the following research question (RQ) is posed:

*RQ: How does the integration of digital therapeutics into the healthcare system change the practices of physicians?*

This study aims to explore how digital therapeutics impact physicians’ roles and tasks, analyzes their integration into clinical practice, identifies barriers to adoption, and discusses implications for workflows and responsibilities. By analyzing the perspectives of German physicians, this research contributes to a deeper understanding of how digital tools shape the healthcare workforce. Our paper is organized as follows: First, we outline our chosen methodology and describe our study sample. Subsequently, we present our results and discuss them afterwards in further detail. In a conclusion, we finally evaluate our study and recapitulate our main findings.

## Methods

The study employs a qualitative approach using semi-structured interviews to explore how digital therapeutics impact physicians in Germany. Germany serves as a particularly relevant context, being one of the first European countries to implement digital therapeutics with a defined reimbursement model through statutory health insurances. Our author team consists of four researchers with extensive expertise in the field of digital health and qualitative interview studies.

### Development of the interview guideline

Our interview guideline was informed by the literature, conversations with a physician and a DTx expert, and discussions within the research team to ensure that all relevant topics were covered consistently across the interviews. The interviews included open-ended questions about physicians’ knowledge of and experience with DTx as well as their thoughts and ideas of how to increase prescription in Germany (see Additional file 1) to encourage participants to share their views openly. While the interview guide included comprehensive questions, it was designed in a semi-structured format to allow for situational follow-up questions. During the initial data collection phase, on the basis of insights from the first interviews, we made minor adjustments to the guide in consultation with the team to further enhance the flow and structure of the interviews as well as the clarity of the questions.

### Recruitment and selection of interview participants

Given our exploratory aim—to understand the overall impact of DTx integration on physicians in Germany—and the diversity of the medical landscape, we decided not to limit our sample to a specific discipline or subgroup but instead aimed for a broad range of perspectives. Therefore, our target group included licensed physicians from different backgrounds and regions, both with and without experience in prescribing DTx. To recruit participants, we collaborated with medical organizations, including a national and several regional medical associations, which disseminated a call for participation through their networks. Physicians contacted us after learning about the study through these channels. In addition, a few respondents were recruited directly through the researchers’ extended professional and private networks.

### Data collection

We collected our data between June 2022 and August 2024 through interviews with 46 physicians across Germany. Toward the end of data collection, many insights began to repeat, and recurring patterns emerged, which indicated data saturation. The length of the interviews varied according to the physician’s available time, experience with DTx and interest in the topic. Most of the interviews lasted between 15 and 20 min, with a minimum of 12 min and a maximum of 35 min. The interviews were conducted by different researchers in one-on-one settings. For the initial interviews, an additional member of the team accompanied the primary interviewer to facilitate follow-up discussion and collective reflection, which informed subsequent interviews. Finally, the research team reviewed the quality of all interviews to ensure consistency.

The interviews were conducted using a variety of modalities, depending on the participants’ preferences. While most interviews were conducted online via Microsoft Teams or Zoom, some were conducted face-to-face or by telephone. All participants were informed of the purpose of the study and the anonymization procedures, and gave their consent to be recorded. The interviewers started with a brief introduction of themselves and the purpose of the study and then followed the semi-structured interview guide. For interviews conducted via Microsoft Teams or Zoom, the built-in transcription feature generated initial transcript drafts. For interviews recorded externally, the A-Train transcription tool was used to produce the first draft. All transcripts were then manually refined to ensure accuracy.

### Final sample

Our final sample consisted of 46 physicians from various disciplines, regions, and levels of experience (see Table 1 for an overview). The majority of participants practiced in the outpatient sector, and the sample primarily included disciplines that are among the main disciplines in Germany (such as internal medicine and general practice) and for which DTx are already available on the market. The gender balance was nearly even, with 21 male and 25 female participants. Physicians’ practical experience ranged from newly qualified to 39 years in practice, ensuring insights from both early career and experienced professionals, with a median of 20–29 years. Table 2 presents the frequencies and distributions of these categories. Since some physicians work in multiple disciplines, the columns for disciplines display absolute values that do not sum to 46. Instead, it illustrates how frequently each discipline is represented among the participants.

**Table 1:** Interview participants

No	m/f	Discipline	Experience (in years)
IP 1	m	P	> 30
IP 2	f	GP	> 30
IP 3	m	OR	20–29
IP 4	f	O	20–29
IP 5	m	I & D	10–19
IP 6	m	GP	> 30
IP 7	m	GP	20–29
IP 8	f	P	> 30
IP 9	m	D	> 30
IP 10	m	D	> 30
IP 11	f	GP	20–29
IP 12	f	GP	5–9
IP 13	m	I & D	20–29
IP 14	f	I, D & GP	20–29
IP 15	m	OR	10–19
IP 16	m	P	> 30
IP 17	f	GP & O	> 30
IP 18	f	GP & I	5–9
IP 19	f	P	10–19
IP 20	f	I	20–29
IP 21	f	P	5–9
IP 22	m	OR	> 30
IP 23	m	GP	> 30
IP 24	f	N	10–19
IP 25	f	GP	10–19
IP 26	m	I & O	20–29
IP 27	m	GP & O	10–19
IP 28	m	N	> 30
IP 29	f	I & O	> 30
IP 30	f	P	20–29
IP 31	m	GP	20–29
IP 32	f	I & D	20–29
IP 33	f	O	> 30
IP 34	f	GP & I	n/a
IP 35	m	I & O	< 5
IP 36	m	GP & I	> 30
IP 37	f	P	20–29
IP 38	f	I & O	> 30
IP 39	f	P	< 5
IP 40	f	GP & O	20–29
IP 41	m	OR	20–29
IP 42	f	N	10–19
IP 43	m	GP & I	20–29
IP 44	m	OR	> 30
IP 45	f	GP	10–19
IP 46	f	I, D & GP	10–19

*D* Diabetologist, *GP* General Practitioner, *I* Internist, *N* Neurologist, *OR* Orthopedist, *P* Psychotherapist, *O* Other

**Table 2:** Sample description

Experience (in years)	Frequency	in %	Disciplines	Frequency
> 30	16	35%	General Practitioner	18
20–29	15	33%	Internist	14
10–19	9	20%	Psychologist	8
5–9	3	6%	Diabetologist	6
< 5	2	4%	Orthopedist	5
n/a	1	2%	Neurologist	3
	*n* = 46	100%	Other disciplines	9

### Data analysis

The interviews were analyzed using qualitative content analysis following the framework outlined by Mayring and Fenzl [24]. We uploaded our transcripts into MAXQDA, which is a widely used software for qualitative analysis purposes. Two researchers of the author team, supported by a student assistant, coded the transcripts and developed a coding scheme through a combined deductive-inductive process. We started with a pre-defined coding scheme, which was based on the topics in our interview guidelines, to have a first structure. In addition, we inductively generated new codes during the analysis process that seemed to be relevant to our research question. Particularly at the beginning, i.e., when coding the first interviews, we had intensive exchanges within the author team and discussed the inductively generated codes. This led us to expand and revise the coding scheme a few times and recode the interviews that had already been coded up to that point. After some rounds of refinement, a coding scheme emerged that we applied to the remaining interviews. Once the coding process was complete, we engaged in a collaborative phase of analysis that involved discussion, collective reasoning and synthesis. On the basis of our coding scheme and the insights we gained from working with the data, we consolidated our key findings by identifying themes and patterns in relation to the overall research question. This process helped us to understand the challenges, benefits and implications of DTx for physicians and summarize, analyze and synthesize the value in the data. While the interviews were conducted and analyzed in German, we translated the quotes in the following results section into English.

## Results

The integration of digital therapeutics (DTx) into the German statutory healthcare system has elicited diverse reactions among physicians. These range from enthusiastic acceptance to skepticism, with many physicians exhibiting a lack of strong opinions or familiarity even three years after DTx were introduced. Despite this variability, the majority of the physicians expressed openness to prescribing DTx, although many had not yet done so. This section outlines the key impacts of DTx integration on German physicians’ practices, covering both current and anticipated changes, as well as short- and long-term implications.

### Searching for information and developing an understanding

The integration of DTx into physicians’ routines requires a solid information base, which many physicians still lack. The interviews revealed that physicians first need to develop awareness of DTx as a treatment option, alongside an understanding of their availability, reimbursement regulations, and suitability for different patient groups. This persistent information gap often delays adoption. Physicians emphasized the importance of familiarizing themselves with the frameworks and conditions surrounding DTx before integrating them into their practices. One participant emphasized the importance of awareness: *“I truly believe that if every doctor was informed that this exists*,* everyone would be happy to prescribe something that they think might help the patient”* (IP 42).

Some physicians built confidence through hands-on experience, testing DTx themselves to assess their usability and identify suitable patient groups. One participant shared, *“We created test accesses for glucura and Una to test them ourselves. We aren’t totally sure yet which patient groups [we could prescribe it to]*,* that means how easy it is to use. We always have to take a look and see to whom we can give it”* (IP 14). This personal engagement allowed for more informed prescribing decisions. Another respondent highlighted the importance of understanding what they prescribe: *“Before I would have prescribed it*,* I would have liked to have looked through it myself. I would have downloaded it and looked at what’s in it. You don’t do that with medication in heroic self-experimentation*,* but you inform yourself via specialist literature and specialist information about certain medications. And I think it has to be the same with an app*,* if I prescribe it*,* then I also have to know what I’m prescribing”* (IP 29).

However, this process is time-consuming and difficult to integrate into daily work. One physician noted, *“But then we have the problem that you really have to spend a lot of time familiarizing yourself with it. You actually have to go through the entire DiGA process. And that’s really time-consuming”* (IP 9). Time was the most frequently cited barrier to acquiring and evaluating information about DTx. Many physicians associated digitalization initiatives in healthcare with inefficiencies and increased workloads, as noted by one respondent: *“For most colleagues*,* digitalization means the telematics infrastructure. Non-functioning e-prescriptions*,* an electronic patient file that only creates work and offers no benefit at all. And then […] they stop thinking*,* because digitalization is bad*,* it only creates work”* (IP 9).

This lack of familiarity also heightened insecurities, particularly regarding potential regress claims. Some physicians noted the absence of reliable, structured information as a significant challenge: *“But there’s already so much and so confusing that it’s not sorted enough. There are so many on the market and then some fall out again. […] Then there are lists again where you have to look at what is authorized. So that doesn’t make it any easier”* (IP 40). The participants stressed the need for trusted, accessible resources and opportunities to exchange experiences with colleagues. However, personal engagement with DTx was not universally deemed necessary, as some physicians felt confident in prescribing DTx without direct testing, which is elaborated further below.

### Assessing patient characteristics and evaluating patient suitability

While some participants likened DTx prescriptions to traditional treatments, the interviews revealed additional layers of evaluation. Physicians noted the need to consider factors beyond standard medical criteria, such as digital competencies, motivation, intellectual readiness, and specific medical conditions. They emphasized that the responsibility for these evaluations lies with the prescriber. One participant explained, *“It’s really important to pay attention to the framework conditions*,* because it’s not suitable for everyone. So anyone who says they don’t have a mobile phone and can’t do anything with it is already out of the picture. So the affinity for such things is one point. Then*,* you can use it as a temporary solution for depressed people or people with burnout. Therapy places are simply few and far between here. Provided that the person is willing. With Zanadio*,* it is important to see what the motivation is like. It is often the case that many people who are overweight find it difficult to become active themselves”* (IP 40). Motivation was consistently identified as a critical factor for success, particularly in the case of DTx, whose use requires sustained effort. One respondent noted, *“It’s difficult to pick out those who are so motivated that you trust them to manage it on their own at home”* (IP 40).

Age was not seen as a barrier, with one physician challenging assumptions about older patients’ abilities: *“It’s always said that [DiGAs] are something for young people*,* for the digitally savvy ones. I have to say quite honestly*,* I think this is complete nonsense*,* because we have a lot of older patients who deal with it really well and make great use of it because they are interested in it and that has nothing to do with age and so on”* (IP 9). This highlights the need for individualized assessments to determine DTx suitability. Physicians emphasized that deeper conversations with patients were often necessary to evaluate their readiness: *“I cannot assess whether they are savvy in the sense of IT nerds. But we asked them whether they think they can deal with such internet programs. And if they answer this positively*,* then this is also an option”* (IP 2). These additional responsibilities add complexity to consultations and impact the physician-patient interaction, which is further explored in the next subsection.

### Redefining the physician‒patient relationship and interaction

The integration of DTx reshapes the physician‒patient relationship by influencing consultation content and dynamics. Physicians noted that prescribing DTx often requires detailed explanations of their functionality, suitability, and expected outcomes. One participant observed, *“[…] we simply don’t have time for the training. We don’t have time for it and*,* of course*,* it’s not compensated. I don’t need to explain to anyone how to take a pill. Even if [I have to]*,* it’s done really quickly. But [prescribing] a DiGA is much more time-consuming. And that’s the problem*,* that this is not compensated”* (IP 9). For some patients, higher levels of guidance and support may be necessary, further extending consultation times. The lack of financial compensation for this additional workload emerged as a recurring frustration among physicians. One respondent highlighted, *“They offered us [to test the DiGA]*,* but we refused. It is simply a time issue”* (IP 7).

Interestingly, discussions about DTx were often initiated by patients themselves. Many physicians noted that patients were becoming more active participants, frequently bringing up specific DTx they had researched: *“Then*,* we look at them together. See if it fits*,* and if we both realize that it suits the patient*,* then I prescribe it at the patient’s request”* (IP 45). However, these new demands often detracted from the limited time available for traditional patient interactions. Physicians stressed the importance of maintaining a balance to ensure that consultation quality was not undermined.

Opinions varied on the level of technical expertise physicians needed for prescribing DTx. Some believed it was sufficient to rely on regulatory checks: *“If I prescribe them*,* then I assume that the health insurance company has checked the app for data protection and that it guarantees the patient that their data is protected within the application”* (IP 29). Others felt that it was necessary to develop a deeper understanding of DTx to communicate effectively with patients: *“How do I communicate it to the patient? […] So first*,* to know what exactly the DiGA actually does and then to communicate it in such a way that these pitfalls*,* so to speak*,* such as not sticking with it and insufficient skepticism*,* are kept to a minimum”* (IP 45).

A related aspect of the physician‒patient relationship involves the perceived shift in responsibility. Some physicians welcomed the way DTx encouraged patients to take greater ownership of their health: *“It makes sense to transfer some of the responsibility to the patient when it comes to monitoring the intake of medication or entering symptoms. It’s nice if the patient has documented this well and you can see trends over time*,* because we only ever have one-off outpatient visits”* (IP 38). Others expressed concerns that reliance on DTx could depersonalize the relationship: *“Perhaps [prescribing a DiGA] could also lead to the fact that sometimes you don’t feel like you are being taken seriously or that there is a plan to somehow get through [the treatment] more quickly or something”* (IP 21).

### Expanding treatment opportunities

Once physicians become aware and informed about DTx, they often recognize how these tools expand their treatment options. Physicians highlighted the value of DTx in addressing gaps in care, such as long waiting times for specialist treatments. One participant emphasized their role in underserved areas: *“But above all in the underserved areas*,* for mental health*,* where there are no psychotherapists*,* a lot more is being done”* (IP 7). DTx were seen as particularly effective for bridging therapy-free intervals: *“This is primarily about bridging the therapy-free interval*,* because we have very long waiting times for therapy places. It is not a substitute for psychotherapy*,* but the patient has the impression that they are already doing something and*,* as I said*,* you can bridge the gap a little bit”* (IP 45).

Physicians agreed that DTx are not standalone treatments but rather part of a multimodal therapeutic approach. As one respondent explained, *“[DiGAs] should be incorporated into the overall program” (IP 26)*. In some cases, DTx prescriptions also have budgetary implications. For example, prescribing a DTx instead of physiotherapy could free up budget allocations for other treatments: *“The main characteristic is that you save on physiotherapy budget”* (IP 41). Physicians also recognized the potential for DTx to provide additional insights into patient health. However, some noted that these data were often inaccessible: *“[…] we need to have access to these data so that we can integrate them into our treatment process”* (IP 9).

### Shifting mindsets and evolving clinical workflows

The integration of DTx into clinical practice introduces the potential for long-term changes in treatment processes and physician mindsets. While some physicians viewed DTx as an incremental adjustment, most acknowledged the significant shifts required in habits, decision-making, and workflows. Physicians highlighted the challenges of adapting to new tools, emphasizing that changes require consistent exposure and reinforcement. One respondent noted, *“As a medical expert*,* you also have your habits*,* and whenever something changes in medicine*,* you first have to get out of your structures. […] So user-friendliness and access to the application must be easy to integrate into everyday life”* (IP 40).

Some physicians expressed reluctance to adopt DTx immediately, citing the presence of established alternatives: *“We have a very wide range of things we can already offer patients. […] And these are well-established and validated things. And before I go into this somewhat murky forest of DiGAs*,* I would actually offer these first”* (IP 5). Over time, positive experiences with motivated patients reinforced confidence in prescribing DTx and underscored their potential to transform treatment workflows. One physician who had embraced DTx early shared how they became a routine part of practice: *“Then*,* we always check the directory to see which app we can prescribe”* (IP 33).

However, the integration of DTx remains a gradual process. Some noted systemic challenges, as one participant explained: *“DiGAs have been deliberately placed by the legislator alongside the treatment and have not become part of the treatment. And therefore*,* they will always have their problems”* (IP 9). Others pointed to the time required for broader adoption: *“Even for pharmaceutical therapies*,* it often takes several years for them to become properly established. This will be no different with DiGAs and will probably take even longer”* (IP 28).

## Discussion

This study examines how the integration of digital therapeutics (DTx) into the German healthcare system, with statutory health insurance reimbursement, impacts physicians’ practices up to three years after their introduction. While the results reveal immediate challenges, they also point to broader implications for physician‒patient relationships, equitable access, and long-term systemic changes. This discussion contextualizes the findings, connects them to broader themes, and outlines directions for future research.

### Immediate challenges in adoption

A key finding of this study was the considerable variability in physicians’ awareness, knowledge, and confidence in prescribing DTx. The participants cited the lack of information, knowledge, and time constraints echoing prior research on barriers to digital health and DTx adoption [6, 7, 12]. Most physicians actively sought or plan to familiarize themselves with DTx in greater depth and wish for additional and reliable information sources—ideally provided automatically by third parties such as manufacturers, pharmaceutical representatives, users, or conferences. Therefore, we believe that targeted educational interventions could be an important starting point for removing this barrier for physicians, and increase awareness and knowledge. Educational workshops to increase digital health literacy in general and on DTx knowledge in particular, the integration of DTx topics into medical conferences, and the reimbursement of consultation time for physicians to assess DTx collaboratively with patients could provide specific suggestions. The reimbursement of medical workflows associated with DTx and the need for specific training are also mentioned by Dahlhausen et al. [7] as potential solutions for better DTx integration on the part of physicians. Such interventions could increase knowledge, interest and/or acceptance,—particularly among physicians with less familiarity or perceived relevance of DTx,—the effectiveness of which could be measured in future research. Long-term strategies, such as incorporating DTx into medical education and clinical guidelines, could further contribute to ensuring sustainable integration [31].

However, it is important to note that while these measures address the lack of information cited by many physicians in our interviews and other studies [7, 12], they do not resolve the broader issue of integrating DTx into the healthcare system. As mentioned in the background section, ongoing debates about the pricing and effectiveness of DTx remain prominent among key stakeholders and health experts and highlight systemic issues. While health insurers express concerns about excessive reimbursement costs for DTx due to high pricing by manufacturers, multiple DTx manufacturers have gone insolvent within the first few years [13, 27]. Additionally, criticism regarding the prescription of DTx with insufficiently proven effectiveness raises further questions about their long-term viability. These broader uncertainties could, even among well-informed physicians on existing DTx and the prescription process, negatively impact their willingness to prescribe DTx. Thus, for both short- and long-term adoption, it is necessary not only to improve physicians’ awareness and financial incentives but also to address structural weaknesses and establish a regulatory framework that ensures sustainable integration. Creating conditions that work for all stakeholders—including physicians, insurers, and manufacturers—will be essential to embed digital innovations effectively into healthcare pathways.

### Changes in the physician‒patient relationship

The results reveal significant shifts in the physician‒patient relationship due to DTx integration. Patients increasingly arrive at consultations informed about specific DTx, shifting the traditional balance of expertise. While some physicians appreciated how DTx empowered patients to take more responsibility for their health, others voiced concerns about managing patients’ expectations and sustaining their engagement. In addition, physicians must balance encouraging patient autonomy with choosing the right patients and providing appropriate oversight, particularly for patients with limited digital literacy or motivation. The findings also indicate that tensions might arise between patients’ expectations of physicians as digital experts and physicians’ own role perceptions. As mentioned above, digital health literacy among physicians in Germany is relatively limited [17]. The need for physicians to develop expertise in the field of digital health interventions is also mentioned by Wattanapisit et al. [32]. Particularly in the early stages of DTx adoption, when physicians have little experience with how patients engage with these tools, the lack of standardized processes—such as monitoring usage, assessing treatment success, and determining appropriate follow-up actions—can create additional pressure and uncertainty. This uncertainty may lead to hesitations in prescribing DTx and could even impact the physician–patient relationship if expectations are misaligned. This tension underscores the need for clear action and communication strategies to manage patients’ expectations while maintaining trust. Future studies could explore these challenges and their potential impact on the physician‒patient relationship, as well as investigate ways to support physicians during this transition phase without adding to their existing workload pressures.

### Equity in access and usage

While DTx are intended to be an accessible therapy option for the broader population [28] and the statutory reimbursement framework removes financial barriers for patients, the study raises some questions about equitable access. First, patients consulting physicians who are unfamiliar with or skeptical about DTx may miss out on potentially beneficial treatment options that other physicians with different perspectives or knowledge levels might have prescribed. Second, physicians’ evaluations of patient suitability are partly subjective and often based on “gut feelings”, which may unintentionally exclude vulnerable populations, such as those with perceived low digital literacy or mental health conditions. These two points align with the idea that physicians are considered gatekeepers in the prescription of DTx [8, 26]. However, even when a DTx is prescribed, patients with insufficient digital literacy or cognitive ability may struggle to use it effectively, which limits the intended benefits. Moreover, as many DTx are available only in German or require access to a mobile device, patients who lack the necessary language proficiency or technical infrastructure are automatically excluded. Thus, while statutory reimbursement ensures formal availability, it does not automatically translate into real-world accessibility or equitable access for all patient groups. This issue raises questions about potential additional inequalities introduced through such digital innovations—technologies that are often expected to improve healthcare accessibility. Future research could explore strategies to address these challenges on multiple levels, such as physician training and patient support systems. For example, providing patients with digital health literacy programs could improve their ability to engage with DTx [30], whereas multilingual instructions could reduce language barriers for non-German-speaking patients. Additionally, standardized tools for assessing patient suitability for DTx could help reduce variability in physicians’ evaluations, ensuring more consistent and fair access to these therapies.

### Expanding treatment options and clinical pathways

DTx were found to expand therapeutic options, particularly by bridging gaps in care, such as long waiting times for psychotherapy. This finding is also in line with previous literature [6]. For example, DTx can empower general practitioners to address conditions traditionally referred to specialists, such as mild-to-moderate depression. However, this shift also raises questions about downstream effects on care coordination and the role of specialists, for instance, whether a digital intervention used by a patient before seeing a specialist could (negatively) influence the specialist’s treatment. Furthermore, while most physicians viewed DTx as complementary tools, their integration into multimodal treatment strategies introduces new complexities. Physicians must evaluate how DTx fit within broader therapeutic contexts, particularly when they are not yet included in clinical guidelines. As clinical guidelines take several years to be updated, other sources of information are needed to provide reliable guidance, as mentioned above. One physician group for whom the expansion of treatment options may have a particularly significant impact is general practitioners (GPs). As the first point of contact for many patients, GPs manage conditions across various specialties within their scope or refer patients to specialists when necessary [29]. Consequently, they are likely to encounter the broadest range of potentially prescribable DTx from multiple disciplines and with different functionalities. This could also mean that they might face the highest information demands and must respond to a diverse set of patient requests for different types of DTx. Given the often limited consultation time and the need for broad knowledge across different therapeutic areas, this additional responsibility could further increase pressure on GPs, who must navigate these new interactions while making informed decisions about patients’ treatment pathways. Therefore, future research could specifically examine how GPs navigate the increasing number of treatment options—something that might simultaneously present both an advantage and a challenge—and how this impacts individual physicians as well as the broader integration of DTx into decision-making within therapeutic contexts.

### Long-term systemic changes

The potential of DTx to drive long-term systemic changes was a recurring theme in the interviews. While some physicians viewed DTx as incremental adjustments, others saw them as requiring significant shifts in clinical workflows, decision-making processes, and professional mindsets. This tension reflects the broader challenge of integrating innovation into established systems, where habitual thinking is deeply ingrained [25]. Overcoming these challenges requires sustained efforts to embed DTx into routine practice, and it must align with broader therapeutic approaches. Cultural shifts within the medical community, supported by consistent education and policy adjustments, will be essential. However, it is important to recognize that physicians’ willingness to prescribe DTx is only one factor in their successful integration and long-term establishment in the healthcare system. As mentioned earlier in this discussion, successful integration requires supportive structural conditions at all levels that facilitate adoption rather than hinder it. The ongoing debates surrounding pricing, effectiveness, and regulatory approval processes highlight that multiple systemic factors still need to be addressed [9, 13, 21]. It should be noted that these challenges and discussions are shaped by the specific conditions of the German healthcare system, in which this study was conducted, where regulatory frameworks may significantly influence how long-term systemic changes unfold. Therefore, future research could conduct comparative studies across countries with different healthcare systems to further explore the factors that facilitate or hinder DTx adoption and to understand how different regulatory frameworks or levels of digital health maturity influence DTx integration. Additionally, involving other relevant stakeholders, such as patients, software developers, and policymakers, could provide a more holistic understanding of the ecosystem needed to support DTx integration. Moreover, longitudinal studies tracking changes in perceptions and practices over time would provide deeper insights into the evolving role of DTx in clinical practice.

### Limitations and critical reflection on data collection

This study provides valuable insights into the integration of digital therapeutics (DTx) into the German healthcare system and their impact on physicians’ practices. At this point, we would like to reflect on our research approach, the interpretation of our findings, and acknowledge some limitations.

Our sample of 46 physicians included a diverse range of specialties, regions, levels of experience with DTx, and years of professional practice, with an almost even gender distribution. This broad dataset provided a strong foundation for answering our research question and identifying overall thematic patterns. Due to the recurring patterns that emerged during data collection, we consider the size and composition of our sample to be sufficiently large and diverse to capture the key points relevant to our study. Nevertheless, our sample cannot fully capture the entire complexity of the German medical landscape or account for all individual backgrounds. Physicians working in specific niche fields, underrepresented specialties, or unique institutional settings may have perspectives or experiences that differ from those captured in this study. However, we believe that expanding our sample would not have fundamentally changed our core findings but rather added further nuances. Our current data already indicate that certain opposing perspectives and different understandings exist among physicians within the identified patterns, which suggests that there might be some underlying differences within the medical community regarding the integration of DTx into medical practices. While an in-depth analysis of these variations was beyond the scope of this study, we see this as a promising avenue for future research, which we intend to pursue. A follow-up study could explore these differences in greater detail, particularly regarding subgroup variations among physicians on the basis of factors such as discipline, region, or gender. To achieve this, we plan to conduct a more in-depth analysis of these differing perspectives within our data. Additionally, the sample could be expanded on the basis of specific selection criteria, such as conducting further interviews with physicians from niche specialties or those with characteristics not assessed in the initial data collection, such as physicians with a migrant background or a specific family status.

Second, as with any qualitative research, potential bias and subjectivity in the data collection and analysis must always be considered when conducting or interpreting such studies. The interviewer’s background, role, and knowledge level can influence both the data collection and the interpretation of findings. While some degree of bias might be inevitable, we took several measures to mitigate its impact. First, all the researchers approached both data collection and data analysis with an open mind and were driven solely by their interest in the research topic (and not, for example, by any commercial interests). The interview guide was informed by the literature and the input of two experts and was developed collaboratively in a team consisting of researchers with extensive knowledge and experience in the areas of digital health and qualitative research methods. Furthermore, interviews were conducted by different researchers, which helped reduce potential interviewer bias and ensured a broader range of perspectives in data collection. Additionally, a second researcher attended the first few interviews to reflect on the process and derive improvements for subsequent interviews. After data collection, all the interviews were reviewed, and no significant differences were identified that might have impacted the findings. Furthermore, while we strived for neutrality in data analysis, individual researchers’ prior knowledge and perspectives could still influence interpretations. To minimize this risk, we maintained a close exchange within our team, particularly during the early coding stages, and derived findings through a collaborative analysis and reasoning process.

Third, the study reflects a specific moment in time—up to three years after the introduction of DTx—when adoption and familiarity with these tools remain in their early stages and differ significantly between individual physicians. Physicians’ attitudes and practices may evolve as DTx become more established, reimbursement frameworks mature, and supporting infrastructures (such as clinical guidelines and educational programs) are implemented. A longitudinal approach or a follow-up study building on the current research could provide deeper insights into how perceptions and practices develop over time. Finally, as already addressed in our discussion, this study was conducted with physicians within the German healthcare system, which operates under unique regulatory and reimbursement models. Therefore, our findings may not be directly transferable to healthcare systems in other countries with different policies, infrastructures, and levels of digital health readiness. Comparative studies across international contexts could help identify universal and context-specific factors influencing DTx adoption and impact physicians’ work practices.

## Conclusion

The integration of digital therapeutics (DTx) into the German healthcare system, supported by statutory health insurance reimbursement, represents a pioneering approach to embedding digital innovations in clinical care. Through semi-structured interviews with 46 physicians from diverse specialties, this study explored the multifaceted impacts of DTx on physicians’ practices three years after their introduction. The findings reveal that DTx influence multiple aspects of medical practice, including information acquisition, patient assessment, physician‒patient relationships, and treatment pathways. While DTx offer new opportunities for bridging gaps in care and enhancing patient autonomy, they also present challenges that require and lead to adjustments in physicians’ workflows, decision-making processes, and professional roles. Physicians highlighted barriers such as the lack of trusted, accessible information, time constraints, and concerns about liability, all of which contribute to varying levels of acceptance and adoption. Furthermore, the need to assess additional patient factors, such as digital literacy and motivation, adds complexity to patient consultations and decision-making. The study also underscores the potential of DTx to transform treatment processes and physician‒patient relationships. By shifting responsibilities toward patients and enabling greater autonomy, DTx align with broader healthcare goals of patient empowerment but may also raise concerns about sustained engagement and equitable access. While DTx are currently viewed as complementary tools, their integration into multimodal care and long-term treatment strategies requires systematic support, including integration in clinical guidelines and evidence-based frameworks. Future research should investigate how physicians’ attitudes toward DTx evolve over time and assess the long-term effects of these tools on care delivery and patient outcomes. Comparative studies across healthcare systems could provide insights into how regulatory frameworks, reimbursement models, and levels of digital health maturity influence adoption.

## Supplementary Information

Supplementary Material 1.

## Data Availability

The dataset used and analyzed during the current study is available from the corresponding author on reasonable request.
