# Supplementary material for: From awareness to integration: a qualitative interview study on the impact of digital therapeutics on physicians’ practices in Germany
Source: BMC Health Serv Res. 2025 Apr 18;25:568. doi: 10.1186/s12913-025-12656-2 (PMC12007376; doi:10.1186/s12913-025-12656-2)
Supplement: Supplementary file 1 — Supplementary Material 1. [file 12913_2025_12656_MOESM1_ESM.pdf]

Interview guide for conducting qualitative interviews with physicians on the prescription of digital health applications

Introduction

- *Thanks to the participants for their participation and time.*
- *Short introduction of the interviewer, the area of research, and the objective of data collection.*
- *Information on the interview process, the recording, data privacy, anonymization, and use of the collected data. Obtaining informed consent.*

Part 1: Demographic questions

- Could you please briefly describe your medical specialty and your tasks?
- For how long have you been working in your profession? What type of medical facility do you work in?
- As how 'digital savvy' would you rate yourself? How do you define this?
- Which gender do you identify with?

Part 2: Main questions

1) Have you already prescribed or recommended digital health applications?

(If **yes**, continue with part **2.A**; if **no**, continue with part **2.B**)

Part 2.A:

2) Which digital health applications have you already prescribed (name and application area)? Why?

3) To what extent do you prescribe digital health applications (daily, weekly, monthly)?

4) To which patients do you prescribe digital health applications?

   Are there patients you would definitely not prescribe digital health applications to?

5) Based on which criteria do you decide to whom you prescribe digital health applications? What role do patient-related factors play in the prescription of digital health applications (e.g., age, psychological factors, etc.)?

6) Do you prescribe digital health applications proactively or upon patient request? Would you be influenced if a patient specifically asked for a digital health application?

7) How would you rate patients' knowledge about digital health applications?

   Do your patients know that this form of treatment exists?

8) How would you rate physicians' knowledge about digital health applications? Are your colleagues aware of digital health applications? If not, what do you think the reasons are? What influences or would influence them to prescribe digital health applications?

9) What role do recommendations from colleagues play for you when prescribing digital health applications?

10) What is your experience regarding patient adherence and consistent use of digital health applications? How do you ensure success in this regard?

11) Have you ever prescribed a digital health application to a patient who was not confident about using it? If so, what was your experience?

12) In general, what has been your experience with prescribing digital health applications? What worked well, what not?

    How does this process differ from prescribing medication? What distinguishes a digital health application from a traditional treatment?

13) How do you get to know about new digital health applications on the market?

    How do you assess whether they are eligible for your patients?

14) Which role do medical guidelines play for you in prescribing digital health applications?

15) Are there situations or indications where prescribing a digital health application would be exactly the wrong thing to do? Which ones?

16) How would you rate the process of prescribing digital health applications to date?

    Do you feel capable of prescribing digital health applications? Do you feel sufficiently informed to prescribe a digital health application? (What if the patient asks about data protection?)

17) What do you wish for the future prescription of digital health applications?

    What needs to happen for digital health applications to be prescribed more frequently?

    How could physicians be trained in working with digital health applications? What learning content would be necessary?

Part 2.B:

2) Why have you not yet prescribed any digital health applications?

3) Could you imagine prescribing digital health applications?

4) To which patients would you prescribe digital health applications?
Are there patients you would definitely not prescribe digital health applications to?

5) Based on which criteria would you decide to whom you would prescribe a digital health application? What role would patient-related factors play in the prescription of digital health applications (e.g., age, psychological factors, etc.)?

6) Would you prescribe a digital health application to a patient if he or she actively asks you for it? Would you be influenced if a patient specifically asked for a digital health application?

7) How would you rate patients' knowledge about digital health applications? Do your patients know that this form of treatment exists?

8) How would you rate physicians' knowledge about digital health applications? Are your colleagues aware of digital health applications? If not, what do you think the reasons are? What influences or would influence them to prescribe digital health applications?

9) What role would recommendations from colleagues play for you when prescribing digital health applications?

10) Which role do medical guidelines play for you in prescribing digital health applications?

11) What do you think distinguishes a digital health application from a traditional treatment?

12) How do you get to know about new digital health applications on the market?

How would you assess whether they are eligible for your patients?

13) Are there situations or indications where prescribing a digital health application would be exactly the wrong thing to do? Which ones?

14) How would you rate the process of prescribing digital health applications to date?

Do you feel capable of prescribing digital health applications? Do you feel sufficiently informed to prescribe a digital health application? (What if the patient asks about data protection?)

15) What do you wish for the future prescription of digital health applications?

What needs to happen for digital health applications to be prescribed more frequently?

How could physicians be trained in working with digital health applications? What learning content would be necessary?

16) What would have to change for you to prescribe digital health applications in the future?

Part 3: Closing

- Are there things related to the topic that I didn’t ask but you would like to add?
- Thank you for the interview!
